# Integrating the effects of latitude and altitude on the spatial differentiation of plant community diversity in a mountainous ecosystem in China

**DOI:** 10.1371/journal.pone.0174231

**Published:** 2017-03-21

**Authors:** Manhou Xu, Li Ma, Yanyan Jia, Min Liu

**Affiliations:** Institute of Geographical Science, Taiyuan Normal University, Jinzhong, China; University of Waikato, NEW ZEALAND

## Abstract

Varying patterns of plant community diversity along geographical gradients are a significant topic in biodiversity research. Here, to explore the integrated effects of latitude and altitude on the plant community diversity in a mountainous ecosystem, we set Guancen Mountain in the northern section, Guandi Mountain in the middle section, and Wulu Mountain in the southern section of the Lvliang Mountains as study areas, and the plant community diversity (basal diameter and height of tree and species diversity indices of shrub and herb) was measured horizontally at different latitude gradients and vertically at different altitude gradients in late July 2015. The results showed that (1) the trees were taller and wider at the middle latitude and higher altitude with a stronger spatial heterogeneity in the structures along the latitudinal and altitudinal gradients. The evergreen tree growth preceded that of the deciduous trees in the higher latitude and lower altitude regions, whereas the deciduous tree growth preceded that of the evergreen trees in the middle latitude and higher altitude regions. (2) Shrubs and herbs tended to grow well in the lower latitude and middle-lower altitude regions. The shrubs had a larger species diversity at lower latitude and lower altitude, but the species diversity of the herbs was not sensitive to the influences of the latitudinal and altitudinal gradients. With the latitude and altitude increasing, perennial herbs tended to grow well at higher latitude and higher altitude, while annual herbs tended to thrive at the middle latitude and lower altitude. In conclusion, environmental deviations caused by latitudinal and altitudinal gradients had great influences on the spatial distributions of the plant community diversity in the Lvliang Mountains.

## Introduction

Biodiversity is not only a fundamental component of ecological studies but also a hot topic in global change research. It has great importance in maintaining the global ecological balance and promoting human sustainable development [1]. Over the last 100 years, the varying patterns of species diversity along geographical gradients has been a significant topic in biodiversity research [2].

For nearly half a century, many scholars at home and abroad have carried out many studies on latitudinal patterns of species richness. They showed that peak values of species richness appeared at middle latitude regions but that there were no significant trends with the latitudinal gradients [3–5]. However, other studies deemed that the species richness tended to decrease with the latitude elevation [6–10]. Many researchers put forward theories or hypotheses to clarify this matter, such as the Rapoport law, energy hypothesis, and middle-domain effect [11, 12], but the conclusions obtained are inconsistent.

The habitat heterogeneity theory concludes that almost no species exist in all habitat types [13]. Therefore, the species diversity has an ascending trend as the number of habitat types increases regionally [14, 15]. At regional scales, the plant species diversity tends to increase from the south to the north, while at community scales, the species diversity presents a decreasing pattern with the latitudinal gradients [4]. This might mean that the latitudinal distribution patterns of species diversity show various manifestations, which are related to different study scales and corresponding ecological control mechanisms to some extent [16]. Thus, in future comparative studies, we should attach importance to latitudinal patterns and the inner mechanisms of diversity at specific sampling scales, which may resolve the inconsistencies between studies [17].

The altitudinal gradient patterns of the plant community diversity are controlled by the vegetation evolvement, species evolution, geographic variation, and environmental factors, which reflect biological and ecological characteristics and the distribution status, as well as adaptation to the environment [18–20]. Many important research achievements were realized regarding the variation patterns of the plant community diversity over altitudinal gradients [21–24]. It was suggested that the community diversity reaches its maximum at middle altitudes, declines gradually with rising altitudes, or has no relationship with the altitude [25].

The altitudinal distribution patterns of the plant community diversity had greater discrepancies in mountainous regions and between different community types, which might be connected to the regional environmental conditions, relative heights of mountains, and geological landscape. Concerning the altitudes of mountains, serious human disturbances had negative effects on the biodiversity in low-altitude regions [26]; in high-altitude regions, a cold climate slowed down plant growth and soil development, while other harsh environments exceeded the tolerance limitations for growth of the majority of species, such as by intense solar radiation or large temperature differences between day and night [27]. In the middle-altitude regions, the species diversity was relatively higher due to less human disturbances and the formation of transition zones of plant species differentiation between the low- and high-altitude regions [27]. Hence, it is of vital scientific significance and value to study the plant community diversity and its altitudinal gradient patterns in mountainous regions under climate change and human disturbances.

Variations in the latitude and altitude in mountainous regions lead to changes in the temperature, humidity, heat and illumination that then affect the plant species composition and community structure. The changes in these environmental factors along the altitudinal gradients were 100 times faster than those along the latitudinal gradients [28–31]. The Lvliang Mountains are located on the Loess Plateau, which is characterized by rare precipitation, intense evaporation, severe soil erosion, and a low ability to resist natural hazards. They are located in a region where the ecological environment is harsh in nature and extremely difficult to recover when destroyed. Previous studies on vegetation in the Lvliang Mountains mostly focused on single mountains, such as Guancen Mountain in the north, Guandi Mountain in the middle, and Wulu Mountain in the south [31, 32]. However, according to the viewpoints and methods of system theory, there is a lack of systematic studies on the vegetation spatial variations that utilize a method considering the Lvliang Mountains as a whole. For this reason, we set Guancen Mountain, Guandi Mountain and Wulu Mountain as study areas to explore the latitudinal and altitudinal gradient patterns of the plant community diversity in the Lvliang Mountains.

## Materials and methods

### Study area

**Fig 1:**
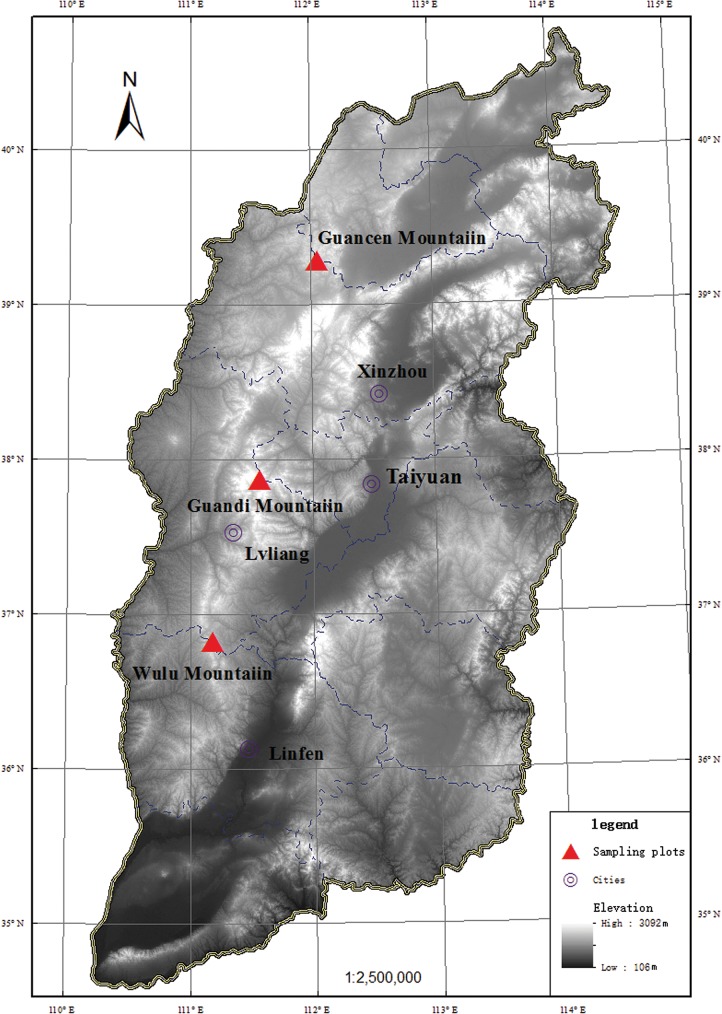
Topographic map of study area. Different degrees of black colour show elevations from 106 m to 3092 m in Shanxi Province. DEM (Digital Elevation Model) data source is USGS EROS (Earth Resources Observatory and Science Center): http://eros.usgs.gov/.

The Lvliang Mountains lie in the west of Shanxi province, with its natural features presented in Table 1. They include Guancen Mountain in the north, Guandi Mountain in the middle, and Wulu Mountain in the south (Fig 1). Guancen Mountain is located in Ningwu county in Xinzhou city, where the vegetation possesses a clear vertical distribution characterized by junctions between broadleaved deciduous forests in the warm temperate zone and grasslands in the temperate zone. Guandi Mountain is located in Jiaocheng county in Lvliang city, where the climate belongs to a sub-humid continental monsoon climate in the warm temperate zone, and the vegetation has an evident vertical distribution with the altitude. Wulu Mountain is located at the junction between Pu and Xi counties in Linfen city, where the climate is a continental monsoon climate in the warm temperate zone, and the vegetation has a distinct transitivity, with the zonal vegetation being broadleaved deciduous forests.

**Table 1:** Natural features of the study area.

Mountain	Coordinates	Elevation ^a^	Mean annual temperature	Mean annual precipitation
Guancen Mountain	38°57′—39°03′ N, 112°36′—112°37′ E	1740–2675 m	6–7°C	450–500 mm
Guandi Mountain	37°20′—38°20′ N, 110°18′—111°18′ E	1800–2460 m	3–4°C	830.8 mm
Wulu Mountain	36°23′—36°38′ N, 111°2′—111°18′ E	1324–1586 m	8.7°C	500–560 mm

^a^ Elevation is for experimental plots, not for the corresponding mountain.

### Experimental design

In the middle ten days of June in 2015, the experimental plots were selected and investigated along the north-south direction of the Lvliang Mountains. Prior to carrying out this experiment, we obtained permissions from the Luyashan National Nature Reserve (Xinzhou city) for Guancen Mountain, from the Pangquangou National Nature Reserve (Lvliang city) for Guandi Mountain, and from the Wulushan National Nature Reserve (Linfen city) for Wulu Mountain. Our field studies did not involve endangered or protected species. First, in the horizontal direction, the Lvliang Mountains were divided into Guancen Mountain, Guandi Mountain and Wulu Mountain at different latitudes to survey the features of vegetation horizontal distribution patterns. Their average latitudes were 38.5° N, 37.5° N and 36° N, respectively. Second, in the vertical direction, different altitudes were selected from each mountain to investigate the properties of the vegetation vertical distribution patterns in the Lvliang Mountains. Five altitudinal gradients were selected on Guancen Mountain, which were 1740 m, 1892 m, 2100 m, 2610 m and 2675 m, and the corresponding natural vegetation types were broadleaved deciduous forests, mixed coniferous broadleaved forests, shrub-grasslands, evergreen coniferous forests, and subalpine meadows. Four altitudinal gradients were selected on Guandi Mountain, which were 1800 m, 1950 m, 2270 m and 2460 m, and the corresponding natural vegetation types were shrub-grasslands, mixed coniferous broadleaved forests, evergreen coniferous forests, and subalpine meadows. Three altitudinal gradients were selected in Wulu Mountain, which were 1324 m, 1370 m and 1586 m, and the corresponding natural vegetation types were shrub-grasslands, shrubwoods, and broadleaved deciduous forests. Finally, quadrats with different areas were sampled on the basis of the plant lifeforms (tree, shrub and herb). The survey area was 1000 m^2^ for deciduous and evergreen trees, with a quadrat size of 20 m×50 m, which was sampled once at each altitude. The survey area was 200 m^2^ for the deciduous and evergreen shrubs, with a quadrat size of 10 m×20 m, which was sampled three times at each altitude. The survey area was 1 m^2^ for the annual, biennial and perennial herbs, with a quadrat size of 1 m×1 m, which was sampled five times at each altitude. The number of quadrats in the experimental plots was 108 in total, and the surveyed plant species are listed in S1 Table.

### Vegetation measurement

In last ten days of July in 2015, the plant community diversity was investigated in the Lvliang Mountains with the altitudes and geographical coordinates being simultaneously recorded by GPS in each plot. For the trees, the quantity of each species was measured in units of plants, and the diameter at breast height and basal diameter were measured at 1.3 m and 0.3 m heights from the ground, respectively, using a steel tap 3 m in length. For the shrubs, the quantity of each species was measured in units of plants or clusters, and the height and crown diameter were measured by box staffs 5 m in length. The herbs were measured in units of plants, and the average height, frequency and coverage were determined. The individual heights from which the average was calculated were measured with a steel tap 3 m in length, and the frequency and coverage were measured by a man-made aluminium quadrat frame with a size of 1 m×1 m whose interior was divided into 100 grids of 10 cm×10 cm in size. A tree was classified into the shrub layer if its height was less than 5 m, and tree seedlings were classified as herbs if they were in the herbaceous layer. When strange species were encountered in the investigation, specimens were collected with specimen holders, and they were brought back to lab, where they were authenticated with plant retrieval lists or by specialists working on plant taxonomy studies. Definite lifeforms of different species were recognized using the book *Flora of China* (http://www.floraofchina.org/) and were described as deciduous or evergreen trees shrubs and as annual, biennium or perennial herbs.

### Data analysis

(1) Calculation of tree height. Eighteen groups of field-measured data from Li et al. [33] were adopted to establish optimal function relation models between the tree diameter at breast height and height. Data included the tree diameter at breast height and height in mountainous regions that were similar to our study areas in arboreal lifeforms. A most significant relationship by a power exponential function existed between the tree diameter at breast height and height, as *y* = 79.559*x*^1.283^ (*R*^2^ = 0.867, *P*<0.001), where *x* was the diameter at breast height (m), and *y* was the height (m). In our research, the tree diameter at breast height and basal diameter were measured in needle-leaved and broad-leaved forests at different latitudes and altitudes in the Lvliang Mountains, and the tree height was calculated indirectly according to the above equation.

(2) Calculation of shrub and herb species diversity. α diversity indices [29–31] were used to analyse the shrub and herb species diversity at different latitudes and altitudes in the Lvliang Mountains, such as the Margalef index, Simpson index, Shannon index and Pielou index, with equations of

$$IV_{\mathrm{Shrub}}=\frac{ra+rh+rc}{3}$$ (1)

$$IV_{\mathrm{Herb}}=\frac{ra+rh+rf+rc}{4}$$ (2)

$$H^{\prime }=1-\sum_{i=1}^{S}p_{i}^{2}$$ (3)

$$H=-\sum_{i=1}^{S}p_{i}^{}\;\ln\left(p_{i}\right)$$ (4)

$$E=\frac{H}{\ln\left(S\right)}$$ (5)

$$p_{i}=\frac{IV_{i}}{IV_{total}}$$ (6)

where *IV* is the important value, *ra* is the relative abundance, *rh* is the relative height, *rf* is the relative frequency, and *rc* is the relative coverage; *H′* is the Simpson index; *H* is the Shannon index; *E* is the Pielou index; *i* is a plant species; and *S* is the Margalef index, which is the sum of all plant species in the quadrat frames of the experimental plots.

(3) Regression relations were first developed by SPSS 16.0 software (SPSS Inc., Chicago, IL, USA) between the tree basal diameter and height to obtain optimal function equations, and regression analysis diagrams were then drawn by Origin 8.1 software (Origin Lab, Northampton, MA). Origin 8.1 software was also used to draw trend charts for the tree basal diameter and height, shrub and herb species diversity and herb important values with latitudinal and altitudinal gradients, and SPSS 16.0 software was also used to conduct significance tests by the one-way analysis of variance (ANOVA) on the tree basal diameter and height and herb important values for different latitudes, altitudes, and plant lifeforms. Multiple comparisons were also performed by using Duncan’s test following a one-way ANOVA.

## Results

### Effects of latitude and altitude on basal diameter and height of trees

**Fig 2:**
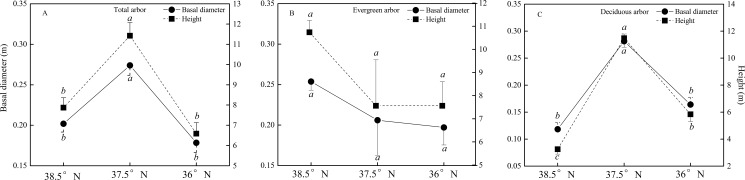
Basal diameter and height of trees at different latitudes in the Lvliang Mountains. A, B and C show the basal diameter and height of all trees, evergreen trees and deciduous trees, respectively; 38.5° N, 37.5° N and 36° N are the latitudes of Guancen Mountain, Guandi Mountain and Wulu Mountain, respectively.

The basal diameter and height of the trees presented unimodal trends of increasing at first and then decreasing with the latitude (Fig 2A), and they were significantly greater at the latitude of 37.5°N than at latitudes of 38.5°N and 36°N (*P*<0.05). The evergreen trees and deciduous trees manifested different trends with the latitude. For the evergreen trees, the basal diameter and height decreased with decreasing latitude, but the differences were not significant (*P*>0.05) (Fig 2B). For the deciduous trees, the basal diameter and height increased at first and then decreased. The basal diameter was significantly greater at 37.5°N than at 38.5°N or 36°N (*P*<0.05), and the height was significantly greater at 37.5°N than at 36°N and at 36°N than at 38.5°N (*P*<0.05) (Fig 2C).

**Fig 3:**
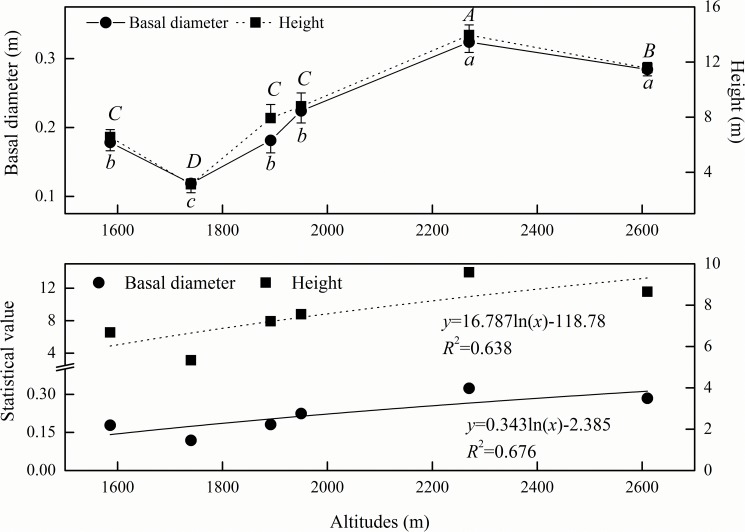
Variations of tree basal diameter and height at different altitudes. The altitude of 1586 m is on Wulu Mountain; 1740 m, 1892 m and 2610 m are on Guancen Mountain; and 1950 m and 2270 m are on Guandi Mountain. Capital and small letters show ANOVA results of height and basal diameter, respectively, at different altitudes.

With the altitude, both the basal diameter and height of the trees increased markedly (*P*<0.05) (Fig 3). For the basal diameter, the altitudes of 2610 m and 2270 m showed the maximal value of 0.304 m, while 1950 m, 1892 m and 1586 m had an average of 0.195 m, and 1740 m had the minimal value of 0.119 m. The differences between these three sets were significant (*P*<0.05). For the height of the trees, there were four values that exhibited significant differences among them (*P*<0.05). At 2270 m, the height was the maximum (13.95 m); at 2610 m, the height was 11.56 m; at 1950 m, 1892 m and 1586 m, the mean height was 7.75 m; and at 1740 m, the height was the minimum (3.12 m).

**Fig 4:**
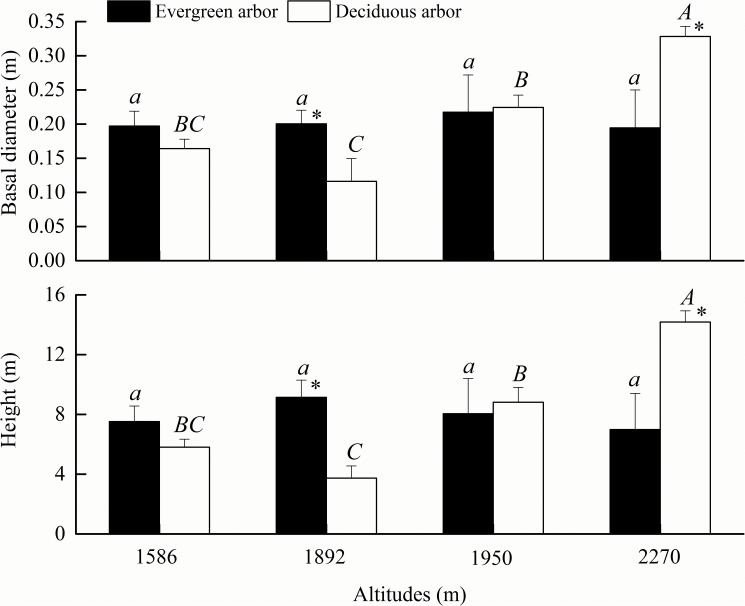
Variations of tree basal diameter and height at different altitudes. The altitude of 1586 m is on Wulu Mountain; 1892 m is on Guancen Mountain; and 1950 m and 2270 m are on Guandi Mountain. Capital and small letters show ANOVA results of deciduous trees and evergreen trees, respectively, at different altitudes. * indicates ANOVA results of deciduous trees and evergreen trees at the same altitude.

There were discrepancies in the tree basal diameter and height variations in different life forms with the changes in altitude (Fig 4). With increasing altitude, the values for the evergreen trees changed more gradually and non-significantly (*P*>0.05), whereas those of the deciduous trees increased significantly (*P*<0.05). The basal diameter and height of the evergreen trees and deciduous trees had inconsistent differences between different altitudes. At altitudes of 1586–1892 m, the basal diameter and height were greater in the evergreen trees than in the deciduous trees, and their differences were significant at 1892 m (*P*<0.05). At altitudes of 1950–2270 m, the basal diameter and height were greater in the deciduous trees than in the evergreen trees, and the differences were significant at 2270 m (*P*<0.05).

### Effects of latitude and altitude on species diversity in shrubs and herbs

**Fig 5:**
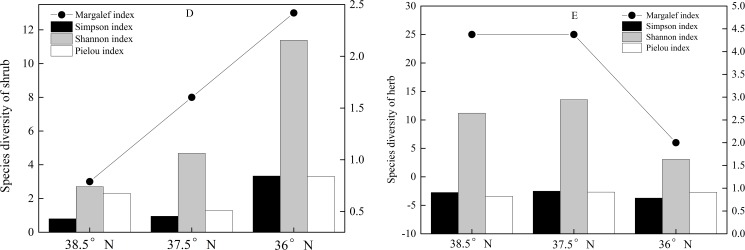
Species diversity indices of shrubs (D) and herbs (E) at different latitudes in the Lvliang Mountains. The latitudes of 38.5° N, 37.5° N and 36° N are for Guancen Mountain, Guandi Mountain and Wulu Mountain, respectively.

The species diversity indices of the shrubs showed an increasing trend with decreasing latitude, except for the Pielou index (Fig 5D). The minimum value of the Pielou index was at the latitude of 37.5°N, with a trend of 36° N>38.5° N>37.5°N, indicating the minimal community stability and the maximal species competition of shrubs being at the middle latitude. The minima of the Margalef index, Simpson index and Shannon index were at the latitude of 38.5° N, and their maxima were at 36° N, manifesting that the species diversity increased with the decreasing latitude. The important values of the evergreen shrubs and deciduous shrubs presented inconsistent trends with the latitude (Table 2). For the evergreen shrubs, the species number and important value tended to increase with the decreasing latitude, and for the deciduous shrubs, the species number tended to increase, while the important value tended to decrease with the decreasing latitude.

**Table 2:** Important values of shrubs and herbs at different latitudes in the Lvliang Mountains.

Latitude ^a^	Evergreen shrubs		Deciduous shrubs		Annual herbs		Perennial herbs	
	Species number	Important value	Species number	Important value	Species number	Important value	Species number	Important value
38.5° N	0	0	3	1	3	0.187	21	0.813
37.5° N	1	0.027	7	0.973	4	0.237	21	0.763
36° N	1	0.083	12	0.917	0	0	6	1

^a^ The Lvliang Mountains are divided into Guancen Mountain in the northern section, Guandi Mountain in the middle section and Wulu Mountain in the southern section. Their average latitudes are 38.5° N, 37.5° N and 36° N, respectively.

The species diversity indices of the herbs showed unimodal trends of first increasing and then decreasing with the latitude. Their maximal values all appeared at the latitude of 37.5°N, and the Margalef index and Shannon index showed greater declines at the latitude of 36° N (Fig 5E). Considering the number and important value of species, the maxima of the annual herbs were at 37.5°N, and the maximal important value of the perennial herbs was at 36° N, but their species number was clearly smaller than those at 37.5°N and 38.5°N (Table 2). The annual herbs were dominant at the middle latitude, while the perennial herbs were dominant at the higher latitude. As a whole, the species diversity indices of the perennial herbs were higher than those of the annual herbs at the different latitudes (Table 3), demonstrating that the perennial herbs dominated. Table 3 reveals that the species diversity indices of the annual herbs realized their maxima at 37.5° N, with a trend of 37.5°N >38.5°N >36° N, while for the perennial herbs, the Simpson index and Shannon index had trends of 37.5°N >38.5°N >36° N, and the Pielou index had a trend of 37.5°N >36° N >38.5°N.

**Table 3:** Species diversity indices of herbs at different latitudes in the Lvliang Mountains.

Latitude ^a^	Simpson index		Shannon index		Pielou index	
	Annual herb	Perennial herb	Annual herb	Perennial herb	Annual herb	Perennial herb
38.5° N	0.267	0.897	0.518	2.521	0.472	0.828
37.5° N	0.65	0.926	1.174	5.559	0.847	1.826
36° N	0	0.785	0	1.634	0	0.912

^a^ The mean latitudes of 38.5° N, 37.5° N and 36° N are for Guancen Mountain, Guandi Mountain and Wulu Mountain, respectively.

**Fig 6:**
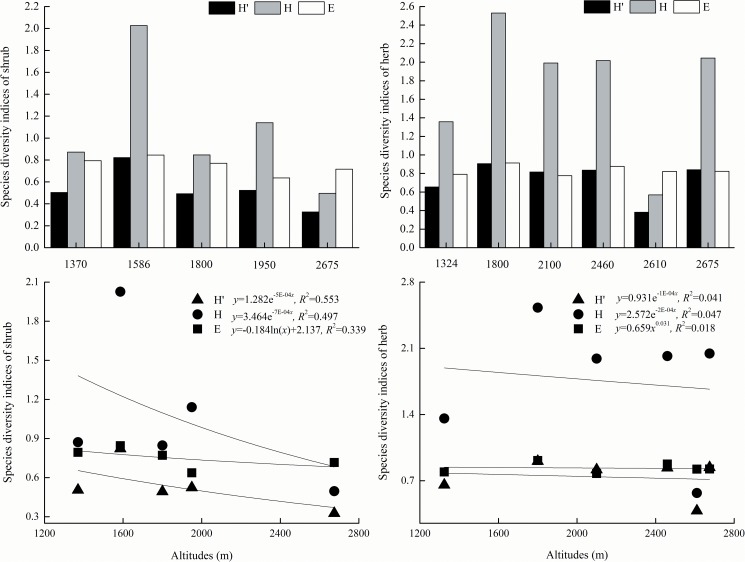
Variations of shrub and herb species diversities at different altitudes. Altitudes of 1370 m and 1586 m are on Wulu Mountain; 1800 m and 1950 m are on Guandi Mountain; and 2675 m is on Guancen Mountain. H′ is the Simpson index, H is the Shannon index, and E is the Pielou index.

The species diversity indices of the shrubs and herbs expressed tendencies of increasing first and then decreasing with the increasing altitude, showing a changing unimodal curve on the low sides (Fig 6). The peak values of all species diversity indices appeared at lower altitudes, 1586 m for the shrubs and 1800 m for the herbs. For the shrubs, the Shannon index was the largest, followed by the Pielou index, and the Simpson index was the smallest at all altitudes except for 2675 m. For the herbs, the Shannon index was the largest, and the Pielou index had only smaller differences from the Simpson index at all altitudes except for 2610 m. The Simpson index and Shannon index of the shrubs decreased significantly with the increasing altitude (*P*<0.05), and the mean determination coefficients of their optimal equations were 0.525. None of the species diversity indices of the herbs had significant differences with the altitude (*P*>0.05), and the mean determination coefficients of their optimal equations were 0.035.

**Fig 7:**
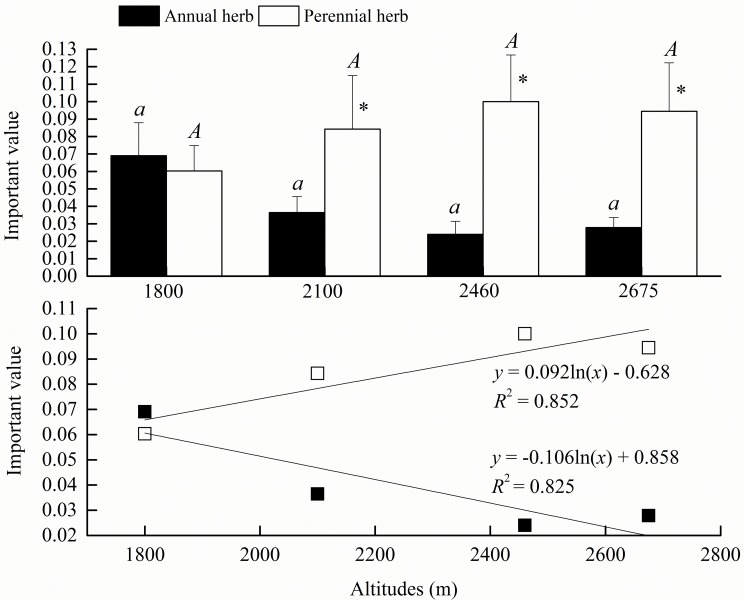
Variations of herb important values at different altitudes. Altitudes of 1800 m and 2460 m are on Guandi Mountain, and 2100 m and 2675 m are on Guancen Mountain. Capital and small letters show ANOVA results of perennial herbs and annual herbs, respectively, at different altitudes. * indicates ANOVA results of perennial herbs and annual herbs at the same altitude.

The important values of herbs presented non-significant differences among the different altitudes (*P*>0.05) but showed the most significant trends with the increasing altitude (*P*<0.001), decreasing significantly for the annual herbs (*R*^2^ = 0.825) and increasing significantly for the perennial herbs (*R*^2^ = 0.852) (Fig 7). The differences in important values differed at various altitudes between the annual herbs and perennial herbs and tended to increase with altitude, with a minimum of 0.01 and a maximum of 0.08. At the altitude of 1800 m, the important value of the annual herbs was slightly greater than that of the perennial herbs (*P*>0.05), but at the altitude of 2100 m, the important value of the perennial herbs was remarkably greater than that of the annual herbs (*P*>0.05). With the increasing altitude, the perennial herbs clearly dominated in the grassland community.

## Discussion

### Integrating the effects of the latitude and altitude on the basal diameter and height of trees

The arboreal growth was greatly influenced by the site conditions, and its distribution pattern was a result of the interactions of various environmental factors, such as the terrain, soil and climate, at different scales [29]. In this research, the basal diameter and height of the trees presented unimodal trends with the latitude increasing in the Lvliang Mountains; the trees grew taller and wider at the middle latitude, and the tree structure had greater spatial heterogeneity between latitudes, with the deciduous trees growing better at the middle latitude, and the evergreen tree growing better at the higher latitude (Fig 2). This might be because the distribution of the arboreal community was affected by the latitude and human disturbances. Regarding latitude, Guancen Mountain, located at a higher latitude in the northern section of the Lvliang Mountains, was subjected to the climate effects of the Mongolian Plateau and had a distinct continental climate. This mountain in the northern sub-region of broadleaved deciduous forests in the warm temperate zone belonged to a transition area between the broadleaved deciduous forests in the warm temperate zone and grasslands in the temperate zone and contained a wide distribution of cold temperate coniferous forests, with *Picea asperata*, *Picea meyeri*, *Picea wilsonii*, and *Larix principis-rupprechtii* being the dominant species [34]. Guandi Mountain at the middle latitude and Wulu Mountain at the lower latitude are in the middle section and southern section, respectively, of the Lvliang Mountains. The two mountains belong to a transition zone from the south to the north sub-region of broadleaved deciduous forests in the warm temperate zone and were mostly covered with natural or artificial broad-leaved forests, such as *Quercus wutaishanica* and *Populus davidiana* [30]. From the view of human disturbances, Guancen Mountain has numerous tourists, as it is a national forest park, but blind exploitation had a certain effect on the natural growth of herbs here. Guandi Mountain and Wulu Mountain possess fine exploitation conditions, so the overcutting was more serious there.

In the Lvliang Mountains, both the basal diameter and height of the trees increased remarkably with the altitude, and the trees grew taller and wider at the higher altitude. The trees had stronger spatial heterogeneity in altitudinal gradient structures, with the evergreen arboreal growth exceeding that of the deciduous trees at the lower altitude and the deciduous arboreal growth superseding that of the evergreen trees at the higher altitude (Figs 3 and 4). This might be the reason why severe human disturbances had negative impacts on the arboreal growth at the lower altitude [26]. The cold climate at the higher altitude resulted in the poor growth of herbs and the slow development of the soil, and the intense solar radiation and great day-night temperature difference exceeded the tolerance limitations of the majority of the herb species, thereby promoting arboreal growth [27]. Hence, the natural environment at different latitudes and altitudes and human disturbances were the principal factors affecting the distribution of the plant community in the Lvliang Mountains. Under the circumstances of no destruction on the patterns of the latitudinal and altitudinal gradients, limiting the human disturbances was an effective measure to protect the arboreal community in the Lvliang Mountains.

### Integrating effects of latitude and altitude on species diversity in shrubs

The species diversity as an essential content of biodiversity is the simplest and most effective method to describe community and regional diversities. Species diversity studies occupy a vital position in studies of whole biodiversity and are critical topics in studies of community ecology. At present, there have been few studies on the variations of the plant species diversity with the latitude at home and abroad. Gentry et al. [35] discovered that the species diversity and richness of the plant community markedly increase with the decreasing latitude. A study by Xie et al. [36] on the plant species diversity in a temperate forest manifested that the species diversity indices of the shrub layer increased continually with the decreasing latitudes in broadleaved deciduous forests. In this research, the species diversity of the shrubs increased at the lower latitude in the Lvliang Mountains (Fig 5), which was consistent with the change features of the latitudinal gradients mentioned above. Many factors impacted the shrub species diversity in the Lvliang Mountains. The primary factors leading to the latitudinal differentiation of the diversity included the temperature, moisture, soil nutrients and succession process [25]. Regions in the lower latitudes of the Lvliang Mountains were rich in water and fertilizer, and the species diversity of shrubs was relatively larger, owing to the diversity of the lower arboreal layer inducing fine development of the shrub layer and fewer human disturbances impacting the shrub layer.

Different types of shrubs showed inconsistent trends at different latitudes in the Lvliang Mountains, where evergreen shrubs tended to grow well at the lower latitude, whereas the deciduous shrubs favoured the higher latitude (Table 2). Among the environmental factors influencing the plant growth, variations of the temperature play an important role. Precipitation increases or decreases caused by seasonal variations generated negative effects on plant growth, which might be partly offset by the temperature enhancement. For temperature variations, the annual mean temperature and monthly mean temperature in January and July had a tendency of increasing from Guancen Mountain to Wulu Mountain in the Lvliang Mountains [32], and this trend played a decisive role in the distribution patterns of different types of shrubs. Consequently, owing to the greater temperature demands for the evergreen shrubs, the evergreen shrubs and deciduous shrubs tended to grow well at the lower latitude on Wulu Mountain and at the higher latitude on Guancen Mountain.

Our research revealed that the species diversity of shrubs and herbs tended to be larger at the middle and lower altitudes in the Lvliang Mountains; the shrub had greater species diversity at the lower altitude, while that of the herbs was not sensitive to the influence of altitude (Fig 6). Li et al. [30] discovered that the overall trends of plant community diversity increased with altitude, with the diversity indices increasing for the shrub layer and decreasing for the herb layer. Qu et al. [27] also obtained that the whole trends of species richness, the Shannon-Wiener index and the Simpson index, were the largest in the herb layer, smaller in the shrub layer and the smallest in the arboreal layer at various levels, indicating that the plant diversity along altitudinal gradients presented a tendency of a unimodal pattern with greater values at the middle altitude. This showed that differences existed not only in the community species diversity of the same plant formation but also in the species diversities of the tree layer, shrub layer, and herb layer in different plant formations.

### Integrating effects of latitude and altitude gradients on species diversity in herbs

Comparisons of the diversity at different levels indicated that the responses of the plant community diversity to the environment were not the same for diverse gradations, and different species exhibited different gradient patterns owing to restrictions from environmental and artificial factors [37–39]. Therefore, the trends of species diversity exhibited differences between the herbs and shrubs. Batunacun et al. [40] discovered that the species diversity of herbs showed a situation of increasing gradually from the north to the south. Sun et al. [41] also determined that the species diversity in the herb layer tended to increase with decreasing latitude. In our research, the distribution patterns of the herb species diversity tended to be the largest at the middle latitude of the Lvliang Mountains (Fig 5). Compared with Guancen Mountain and Guandi Mountain, Wulu Mountain at the lower latitude of the Lvliang Mountains had distinctly reduced altitude and lay in the continental monsoon subhumid climate region of the warm temperate zone, where it was suitable for the growth of secondary forests and shrub vegetation, but the vegetation growth in the herb layer was restricted, making it a diversity centre of the herb layer skewing to Guandi Mountain at the middle latitude in the Lvliang Mountains. This showed that the plant species diversity in the herb layer changed with the latitude, while being affected by complicated habitat conditions such as the altitude and temperature.

The important values of herb in different life forms showed different trends with latitudinal gradients in the Lvliang Mountains in our research; annual herbs were dominant at the relatively middle latitude, while perennial herbs were dominant at the relatively higher latitude; but species diversity indices of perennial herbs were higher than annual herbs at different latitudes (Tables 2 and 3). At present, it is widely believed that the formation of herbs in different life forms was principally impacted by precipitation, whereas in the similar rainfall conditions, water, heat and light conditions need to be considered, which chiefly included average annual precipitation, accumulated temperature and illumination time [42]. From the north to the south in the Lvliang Mountains, the increments of the average annual precipitation increased the number of species and components of the annual herbs, while the hydrothermal matching requirements of Guandi Mountain at the middle latitude were preferred for annual herb growth by comparison with Guancen Mountain at the higher latitude [42]. However, considering whole mountains, the Lvliang Mountains located in the continental monsoon climate region of the warm temperate zone had four distinctive seasons with drought and windiness in the spring and a quick rise of air temperature and had larger diurnal temperature difference. These conditions conformed to the habitat features of perennial herbs. Hence, the hydrothermal distribution status affected by latitude determined the latitudinal distribution patterns of herbs in the Lvliang Mountains.

There were lots of factors influencing community diversity, such as plant community types, altitudes, human disturbance, successional stages, and habitat discrepancy [30]. Herbs in different life forms demonstrated prominent and inconsistent trends at various altitude gradients in the Lvliang Mountains, where perennial herbs tended to grow well at the relatively higher altitude, and annual herbs tended to the relatively lower altitude (Fig 7). This was attributed to plants in the herb layer not only being entirely affected by the altitudinal pattern but also being impacted by canopy density, logging soil, and local microenvironment [19, 27]. The canopy density had distinct effects on the herb distribution by discrepancies from illumination, local humiture, and ultraviolet intensity, where a lower diversity in the tree layer induced good development in the shrub layer and thus limited herb layer growth [19]. We obtained the same results as Qu et al. [27], showing that the altitudinal gradients of the plant species diversity presented an approximately unimodal pattern, with the species diversity being greater at the middle altitude in the Lvliang Mountains. This was because areas of mountainous regions were larger at the relatively lower altitude in the Lvliang Mountains, where their climate included drought and high temperature, so only species that tolerated drought, high temperature, and soil depletion could survive [20, 26].

Serious human disturbances also had adverse impacts on the biodiversity at the lower altitude [26]. For example, human exploitation had a long history in the middle and southern sections of the Lvliang Mountains, which affected the local natural vegetation significantly. Li et al. [30] concluded that the southern section of the Lvliang Mountains had large degrees of crushing in population distribution areas and low population quantity and density owing to stronger human disturbances, so the diversity was seriously threatened in this region. From the northern Guancen Mountain to the southern Wulu Mountain in the Lvliang Mountains, our research obtained that the latitudes and altitudes decreased, with their gradients being not obvious, the vegetation types reduced, and the plant life forms became simple; the tree density increased, but the height increased first and then decreased; the important values of the shrubs and herbs decreased, and their dominant species were distributed at lower locations. Consequently, the responses of the plant species diversity were sensitive to the altitude in the Lvliang Mountains, with the maximal species diversity appearing at the middle altitude. The distribution patterns of the species diversity with the altitude expressed a monotonic function between diversity and resource productivity.

## Conclusion

Under the dual effects of climatic change and human activities, the effects of the latitude and altitude decreased, the number of vegetation types was reduced, and the plant life forms became simple from the northern Guancen Mountain to the southern Wulu Mountain in the Lvliang Mountains.

(1) The trees were taller and wider at middle latitude and higher altitude, with stronger spatial heterogeneity in structures along the latitudinal and altitudinal gradients. The evergreen arboreal growth preceded that of the deciduous trees in the higher latitude and lower altitude regions, whereas the deciduous arboreal growth preceded that of the evergreen trees at the middle latitude and higher altitude regions.

(2) Shrubs and herbs tended to grow well at lower latitude and middle-lower altitude. Shrubs had greater species diversity at lower latitude and lower altitude, but the species diversity of the herbs was not sensitive to the influences of latitude and altitude. With the increasing latitude and altitude, the perennial herbs tended to grow well at higher latitude and higher altitude, while the annual herbs tended to grow well at middle latitude and lower altitude.

## Supporting information

S1 Table Surveyed plant species in study area. Definite life form and Latin name of each species were recognized using the book *Flora of China* (http://www.floraofchina.org/).
(DOC)
