# Correction: Integrating the effects of latitude and altitude on the spatial differentiation of plant community diversity in a mountainous ecosystem in China

**DOI:** 10.1371/journal.pone.0176866

**Published:** 2017-04-26

**Authors:** The PLOS ONE Staff

There are errors in the Funding section. The correct funding information is as follows: This study was financially supported by the National Natural Science Foundation of China (grant 41501219; http://www.nsfc.gov.cn; MHX), the Applied Basic Research Project of Shanxi Province (grant 2016021136; http://www.sxinfo.gov.cn; MHX), the Philosophy and Social Sciences Planning Project of Shanxi Province (No. 3 document in 2015; http://www.sxskw.org.cn; MHX), the Higher School Science and Technology Innovation Project of Shanxi Province (No. 4 document in 2016; http://www.sxedu.gov.cn; MHX), and the Higher School Key Discipline Construction Project of Shanxi Province (No. 4 document in 2016; http://www.sxedu.gov.cn; MHX). The funders had no role in study design, data collection and analysis, decision to publish, or preparation of the manuscript. The publisher apologizes for the errors.
