# Supplementary material for: Integrating the effects of latitude and altitude on the spatial differentiation of plant community diversity in a mountainous ecosystem in China
Source: PLoS One. 2017 Mar 21;12(3):e0174231. doi: 10.1371/journal.pone.0174231 (PMC5360330; doi:10.1371/journal.pone.0174231)
Supplement: S1 Table — Definite life form and Latin name of each species were recognized using the book Flora of China (http://www.floraofchina.org/). (DOC) [file pone.0174231.s001.doc]

**S1 Table. The surveyed plant species in study area.** Definite life form and Latin name of each species were recognized with a book named Flora of China (http://www.floraofchina.org/).

| **Mountains** | **Altitudes** | **Species** | **Life forms** |
| --- | --- | --- | --- |
| Guancen Mountain | 2675m | *Carex asperifructus* Kukenth. | Perennial herb |
|  |  | *Carex lancifolia* C. B. Clarke | Perennial herb |
|  |  | *Elymus dahuricus* Turcz. | Perennial herb |
|  |  | *Poa annua* L. | Annual herb |
|  |  | *Thalictrum petaloideum* L. | Perennial herb |
|  |  | *Ranunculus japonicus* Thunb. | Perennial herb |
|  |  | *Potentilla chinensis* Ser. | Perennial herb |
|  |  | *Potentilla fruticosa* L. | Deciduous shrub |
|  |  | *Rheum franzenbachii* Munt. | Perennial herb |
|  |  | *Polygonum viviparum* L. | Perennial herb |
|  |  | *Aster tataricus* L. f. | Perennial herb |
|  |  | *Leontopodium leontopodioides* (Willd.) Beauv. | Perennial herb |
|  |  | *Carum carvi* L. | Biennial herb |
|  |  | *Oxytropis aciphylla*Ledeb. | Deciduous shrub |
|  | 2610m | *Picea asperata* Mast. | Evergreen arbor |
|  |  | *Ligularia fischeri* (Ledeb.) Turcz. | Perennial herb |
|  |  | *Aconitum carmichaeli* Debx. | Perennial herb |
|  | 2100m | *Geum japonicum* Thunb. var. *chinense* F. Bolle | Perennial herb |
|  |  | *Fragaria orientalis* Lozinsk. | Perennial herb |
|  |  | *Potentilla fruticosa* L. | Deciduous shrub |
|  |  | *Sanguisorba officinalis* L. | Perennial herb |
|  |  | *Linaria vulgaris* Mill. | Perennial herb |
|  |  | *Pedicularis shansiensis* Tsoong. | Perennial herb |
|  |  | *Medicago arabica* (L.) Huds. | Annual herb |
|  |  | *Oxytropis coerulea* (Pall.) DC. | Perennial herb |
|  |  | *Geranium wilfordii* Maxim. | Perennial herb |
|  |  | *Tripolium vulgare* Nees | Annual herb |
|  |  | *Artenmisia gmelinii* Web.ex Stechm | Perennial herb |
|  |  | *Adiantum capillus-veneris* L. | Perennial herb |
|  |  | *Hippophae rhamnoides* L. | Deciduous shrub |
|  |  | *Lithospermum erythrorhizon* Sieb. et Zucc. | Perennial herb |
|  |  | *Saxifraga stolonifera* Curt. | Perennial herb |
|  | 1892m | *Picea asperata* Mast. | Evergreen arbor |
|  |  | *Larix principis-rupprechtii* Mayr. | Deciduous arbor |
|  |  | *Pinus tabuliformis* Carr. | Evergreen arbor |
|  |  | *Betula albosinensis* Burk. | Deciduous arbor |
|  |  | *Betula platyphylla* Suk. | Deciduous arbor |
|  | 1740m | *Populus davidiana* | Deciduous arbor |
|  |  | *Betula platyphylla* Suk. | Deciduous arbor |
| Guandi Mountain | 1800m | *Poa annua* L. | Annual herb |
|  |  | *Festuca ovina*L. | Perennial herb |
|  |  | *Elymus dahuricus* Turcz. | Perennial herb |
|  |  | *Cleistogenes chinensis* (Maxim.) Keng. | Perennial herb |
|  |  | *Scorzonera austriaca*Willd. | Perennial herb |
|  |  | *Artemisia tanacetifolia* Linn. | Perennial herb |
|  |  | *Artenmisia gmelinii* Web. ex Stechm. | Perennial herb |
|  |  | *Leontopodium stoechas* Hand. -Mazz. | Deciduous shrub |
|  |  | *Plantago depressa* Willd. | Annual herb |
|  |  | *Rorippa indica* (L.) Hiern. | Annual herb |
|  |  | *Polygonum aviculare*L. | Annual herb |
|  |  | *Oxytropis ochrocephala*Bunge. | Perennial herb |
|  |  | *Thalictrum petaloideum*L. | Perennial herb |
|  |  | *Gentiana scabra* Bunge. | Perennial herb |
|  |  | *Myosotis silvatica*Ehrh. ex Hoffm. | Perennial herb |
|  |  | *Potentilla fragarioides* L. | Perennial herb |
|  |  | *Carex capillaris* Linn. | Perennial herb |
|  |  | *Ribes mandshuricum*(Maxim.) Kom. | Deciduous shrub |
|  |  | *Hippophae rhamnoides* Linn. | Deciduous shrub |
|  |  | *Potentilla fruticosa* L. | Deciduous shrub |
|  | 1950m | *Potentilla glabra* Lodd. | Deciduous shrub |
|  |  | *Spiraea pubescens* Turcz. | Deciduous shrub |
|  |  | *Ribes mandshuricum*(Maxim.) Kom. | Deciduous shrub |
|  |  | *Artemisia capillaris* | Perennial herb |
|  |  | *Salix linearistipularis* | Deciduous shrub |
|  |  | *Syzygium oblancilimbum* Chang et Miau. | Deciduous shrub |
|  |  | *Caragana acanthophylla* Kom. | Deciduous shrub |
|  |  | *Larix principis-rupprechtii* Mayr. | Deciduous arbor |
|  |  | *Pinus tabulaeformis* Carr. | Evergreen arbor |
|  |  | *Salix matsudana* | Deciduous arbor |
|  |  | *Populus davidiana* | Deciduous arbor |
|  |  | *Betula platyphylla* Suk. | Deciduous arbor |
|  | 2270m | *Larix principis-rupprechtii* Mayr. | Deciduous arbor |
|  |  | *Picea asperata* Mast. | Evergreen arbor |
|  | 2460m | *Stemmacantha uniflora* (L.) Dittrich. | Perennial herb |
|  |  | *Cirsium setosum* (Willd.) MB. | Perennial herb |
|  |  | *Aster tataricus* L. f. | Perennial herb |
|  |  | *Saussurea pectinata* | Perennial herb |
|  |  | *Plantago depressa* Willd. | Annual herb |
|  |  | *Carex asperifructus* Kukenth. | Perennial herb |
|  |  | *Carum carvi* L. f. *gracile* (Lindl.) Wolff. | Perennial herb |
|  |  | *Carex lancifolia* C. B. Clarke | Perennial herb |
|  |  | *Elymus dahuricus* Turcz. | Perennial herb |
|  |  | *Saxifraga stolonifera* Curt. | Perennial herb |
|  |  | *Potentilla anserina* L. | Perennial herb |
|  |  | *Geranium wilfordii*Maxim. | Perennial herb |
| Wulu Mountain | 1324m | *Artemisia argyi* Levl. | Perennial herb |
|  |  | *Artemisia subulata* Nakai. | Perennial herb |
|  |  | *Artenmisia gmelinii* Web. ex Stechm. | Perennial herb |
|  |  | *Taraxacum mongolicum* Hand. –Mazz. | Perennial herb |
|  |  | *Oxytropis coerulea* | Perennial herb |
|  |  | *Swainsonia salsula* Taubert. | Perennial herb |
|  |  | *Lespedeza floribunda* Bunge. | Deciduous shrub |
|  |  | *Carex capillaris* Linn. | Perennial herb |
|  |  | *Carex lanceolata* Boott. | Perennial herb |
|  |  | *Potentilla longifolia* Willd.ex Schlecht. | Perennial herb |
|  |  | *Stipa capillata* Linn. | Perennial herb |
|  |  | *Aconitum barbatum* Pers. var. *puberulum* Ledeb. | Perennial herb |
|  |  | *Humulus japonicus* | Perennial herb |
|  | 1370m | *Rosa xanthina* Lindl. | Deciduous shrub |
|  |  | *Celtis bungeana* Bl. | Deciduous shrub |
|  |  | *Hippophae rhamnoides* Linn. | Deciduous shrub |
|  | 1586m | *Pyracantha fortuneana* (Maxim.) Li. | Evergreen shrub |
|  |  | *Rosa xanthina* Lindl. | Deciduous shrub |
|  |  | *Cotoneaster acutifolius* Turcz. | Deciduous shrub |
|  |  | *Cotoneaster submultiflorus* Popov. | Deciduous shrub |
|  |  | *Cerasus tomentosa* (Thunb.) Wall. | Deciduous shrub |
|  |  | *Rubus parvifolius* L. | Deciduous shrub |
|  |  | *Padus racemosa* (Lam.) Gilib. | Deciduous shrub |
|  |  | *Celtis bungeana* Bl | Deciduous shrub |
|  |  | *Ulmus macrocarpa* Hance. | Deciduous shrub |
|  |  | *Ulmus pumila* L. | Deciduous shrub |
|  |  | *Euonymus alatus* (Thunb.) Sieb. | Deciduous shrub |
|  |  | *Quercus wutaishansea* Mary. | Deciduous arbor |
|  |  | *Acer buergerianum* Miq. | Deciduous shrub |
|  |  | *Syringa reticulata* (Blume) Hara var. *amurensis* (Rupr.) Pringle. | Deciduous shrub |
|  |  | *Pinus bungeana* Zucc. | Evergreen arbor |
